# Propylene Glycol and Maize Grain Supplementation Improve Fertility Parameters in Dairy Cows

**DOI:** 10.3390/ani10112147

**Published:** 2020-11-18

**Authors:** Robert Mikuła, Ewa Pruszyńska-Oszmałek, Paweł Antoni Kołodziejski, Włodzimierz Nowak

**Affiliations:** 1 Department of Animal Nutrition, Poznań University of Life Sciences, Wołyńska 33, 60-637 Poznań, Poland; wlodzimierz.nowak@up.poznan.pl; 2 Department of Animal Physiology, Biochemistry and Biostructure, Poznań University of Life Sciences, Wołyńska 35, 60-637 Poznań, Poland; ewa.pruszynska@up.poznan.pl (E.P.-O.); pawel.kolodziejski@up.poznan.pl (P.A.K.)

**Keywords:** dairy cows, transition period, propylene glycol, maize grain, blood indices, fertility

## Abstract

**Simple Summary:**

The excessive mobilization of fatty acids from dairy cows’ adipose tissue increases blood non-esterified fatty acid concentrations and could have a negative effect on the fertility parameters and milk yield, as well as increase the risk of metabolic disorders and also result in early-lactation culling risk. Propylene glycol and rumen-protected starch from maize grain are commonly used as glucose precursors reducing nonesterified fatty acid levels; however, no such comparisons are available, thus it was decided to assume it as the aim of this study. Propylene glycol had a positive effect on shortening the period to first ovulation. Propylene glycol and maize grain improved the first service conception rate and decreased the number of services per conception in cows. In conclusion, both treatments with propylene glycol and maize grain had a slight effect on the metabolic profile and no effect on milking performance, yet they improved fertility parameters, which could indirectly enhance milk production economics.

**Abstract:**

The aim of the study was to determine the effect of propylene glycol and maize grain content by-pass starch supplementation during the transition period and the first 56 days of lactation on blood metabolic indices, milk production and fertility parameters in dairy cows. Seventy-five Polish Holstein-Friesian dairy cows were assigned to treatment 21 days before calving. The treatments included: TG—2.5 kg triticale grain/cow per day supplemented from 14 days prepartum to day 56 postpartum, PG—2.5 kg triticale grain/cow per day supplemented from day 14 before parturition to day 56 postpartum, and 400 g propylene glycol/cow per day from 14 days prepartum to 14 days of lactation and MG—2.5 kg maize grain/cow per day supplemented from day 14 before parturition to day 56 postpartum. PG and MG had an effect resulting in the highest glucose concentration at 28 d of lactation. Cows assigned to the PG and MG groups had significantly higher cholesterol levels confronted with TG group at day 14 of lactation, while at days 28 and 56 the same difference was observed only between the MG and TG groups. PG had an effect on shortening the period to first ovulation. PG and MG improved the first service conception rate and decreased the number of services per conception in cows. In conclusion, both treatments of dairy cows with PG and MG improved their fertility parameters, while they had a slight effect on their metabolic profile and no effect on their milking performance.

## 1. Introduction

During the past decades intensive genetic selection of dairy cows, which main goal was to increase milk yield, has brought about a level of production in which nutrition allowances are difficult to supply [1]. Nutritional requirements rise rapidly during the last week of pregnancy, similarly as it is the case at increasing milk production after calving, resulting in the energy deficit postpartum [2]. Therefore a higher frequency of metabolic disorders, as well as lower milk yields and inferior reproductive performance are increasingly common [3,4,5] and may result in early lactation culling risk [6]. Many authors claimed that the main problem is nutrition during the transition period and difficulty with meeting nutrient requirements, especially energy [7,8]. In effect cows are affected by excessive lipid mobilization from the adipose tissue, lower appetite and glucose concentration, therefore the risk of ketosis and fatty liver frequency is higher. Incidence of subclinical ketosis can range from 6.9 to 43% [9]; in turn, Berge and Vertenten noted 39% of cows on a European dairy farm classified as having ketosis [10]. Costs connected with ketosis include treatment cost, decreased milk production, inferior reproductive performance, increased culling risk as well as an increased risk of other diseases [7]. Grummer [11] suggested that good preventive measures against ketosis might involve increasing the complete hepatic NEFA oxidation. To date it has not been definitely established how to increase blood glucose concentration, as a factor causing a complete oxidation of NEFA, is a frequently asked question and thus it has been an objective of many studies on glucogenic additives [7,12,13,14,15,16]. It is of importance both for pure science and practice to specify which glucose precursor is better: whether it is the propionate in the rumen either from propylene glycol or excessive degradation of starch, or starch digestion in the small intestine to glucose.

Propylene glycol (PG) has been applied in ketosis treatment since the 1950s [17] and is still in use today [18,19]. It may be used to reduce the negative energy balance after calving and decrease the risk of ketosis and fatty liver [7]. The main part of propylene glycol is metabolized in the rumen to propionic acid and lactic acid, which is the major substrate for hepatic gluconeogenesis in ruminants [20]. Propionic acid is also a main product of microbial degradation of starch from cereal grains, which undergo excessive fermentation in the rumen. Starch from different cereal grains is characterized by unequal rumen microbial degradability [21]. Undegradable starch could be digested postruminally, with most of digestion taking place in the small intestine [22]. Maize starch may be less degradable in the rumen. Digestion of starch in the intestine compared to fermentation and absorption of organic acids in the rumen is more efficient energetically [23]. Correa et al. observed that starch derived from dried maize grain is an important energy source for ruminants [24]. Additionally, Larsen and Kristensen confirmed that glucose absorption in the small intestine is an efficient source of glucose to the peripheral tissues in dairy cows during very early lactation [25]. In turn, Garnsworthy et al. concluded that rumen digestible starch and rumen bypass starch can be equally effective for maintaining plasma insulin and ovarian function of dairy cows in early lactation [26].

Thus, we hypothesized that propylene glycol and/or maize grain contain by–pass starch, which digestion in the small intestine to glucose exerts a better glucogenic effect compared to excessive rumen degradation of starch, while it might inhibit lipogenesis as well as improve the metabolic status, milking performance and fertility. Therefore, the aim of the study was to determine the effect of propylene glycol and triticale grain as propionic acid sources in the rumen and maize grain content by-pass starch supplementation during the transition period and the first 56 days of lactation on metabolic profile indices, milk production and fertility parameters in dairy cows.

## 2. Materials and Methods

All animal procedures were performed in accordance with the guidelines of the Polish Council of Animal Care. The protocol for this study was approved by the Local Animal Care Committee no. 10 of the Poznan University of Life Sciences.

### 2.1. Animal Management, Experimental Design and Diets

Seventy-five Polish Holstein-Friesian dairy cows were assigned to treatment 21 days before the expected calving date (estimated pregnancy period—280 days). Primiparous cows (seven per each group) were divided according to calving date, while multiparous cows (18 per each group) were divided with respect to calving date, parity (2–4), prior milk production (10,398 kg/305 d lactation), body weight and body condition score. The treatments included: TG (2.5 kg triticale grain/cow per day supplemented from 14 days prepartum to day 56 postpartum), PG (2.5 kg triticale grain/cow per day supplemented from day 14 before parturition to day 56 postpartum, and 400 g propylene glycol/cow per day from 14 days prepartum to 14 days of lactation), MG (2.5 kg maize grain/cow per day supplemented from day 14 before parturition to day 56 postpartum). The nutritional values of the feed components were calculated on the basis of the analyzed nutrient contents using the PrevAlim 3.23 software (Educagri/INRA, Theix, France). The diets were balanced according to the French INRA system recommendations (INRAtion 3.3 software, Educagri/INRA, Table 1). Cows were fed a total mixed ration (TMR), which was served to the animals twice a day. Maize, triticale and barley grains were ground. During the study individual voluntary dry matter intake was monitored and recorded daily.

### 2.2. Sample Collection and Analytical Methods

Weekly forage, concentrate and TMR samples were composite for monthly analysis by wet chemistry for crude protein (CP, method 976.05, AOAC [27]), neutral detergent fiber (NDF, PN-EN ISO 16472, [28]), acid detergent fiber (ADF, PN-EN ISO 13906, [29]), calcium (Ca, method 968.08, AOAC, [27]) and phosphorus (P, PN-EN ISO 6491, [30]) and verified with the estimated value.

The body condition score (BCS) was performed according to the methodology presented by Edmonson et al. [31] during the far-off period (−56 and −21 day), on the parturition day and on 14d and 56 day of lactation.

Blood sampling was performed (four hours after morning feeding) 3 weeks and 1 week before calving and on days 14, 28 and 56 of lactation. Samples were collected into tubes with polystyrene separating granules covered with a clot activator, rotated, frozen and stored (−20 °C) for further analyses. Serum was thawed and analyzed for the concentration of nonestrified fatty acids (NEFA), according to Duncomb’s colorimetric method [32]. The concentrations of glucose (G 7518-400), cholesterol (C7509-400) and triglycerides (T7531-400), blood urea nitrogen (B7550-400), as well as the activity of aspartate aminotransferase (ASPAT) (A7560-400) were analyzed applying a Pointe Scientific (Canton, MI, USA) reagent. Absorbance of the incubated samples was measured using a Marcel Media spectrophotometer (Marcel S.A., Zielonka, Poland) and a Hellma microcuvette (Hellma GmbH & Co., Müllheim, Germany).

The cows were milked twice a day, individual milk yields were recorded daily during 56 days of lactation. Milk samples were collected during milkings twice a day (morning and afternoon) at weekly intervals. Samples were collected into tubes with 2-bromo-2-nitropropane-1,3-diol, next refrigerated and delivered to a commercial laboratory (Milk Quality Laboratory, the Polish Federation of Cattle Breeders and Dairy Farmers, Krotoszyn, Poland) for analyses of fat, protein and lactose. Energy corrected milk (ECM) was calculated according to Reist et al. Reist et al. (2003) as [(0.038 × g crude fat + 0.024 × g crude protein + 0.017 × g lactose)] × kg milk/3.14 [33].

Fertility parameters, such as days to the first ovulation, first-service conception rate, services per conception and day open, were also recorded. The cows were observed visually around the estrus period and the first ovulation was confirmed using an ultrasound scanner equipped with a 7.5 MHz convex transducer (Pie Medical Scanner 200; Pie Medical Imaging BV, Maastricht, The Netherlands). During the study cows’ health status was monitored.

### 2.3. Statistical Analysis

The obtained results were processed statistically using the SAS 9.1 [34] SAS^®^/STAT software package (SAS Institute Inc., Cary, NC, USA). One-way analysis of variance was conducted using the GLM Procedure and the Duncan test. Significance was declared at *p* ≤ 0.05.

## 3. Results

Propylene glycol and maize grain had no effect on BCS and condition changes during the last 21 d of the dry period as well as during the first 2 months of lactation (*p* > 0.05, Table 2).

**Figure 1:**
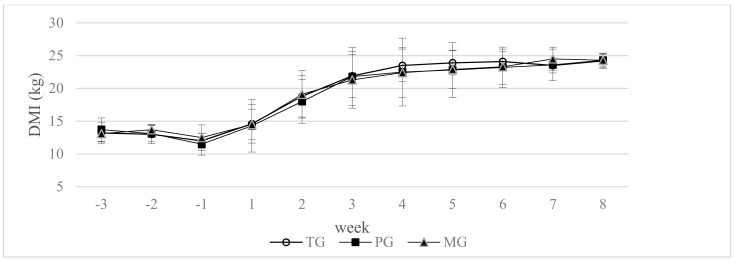
Effect of propylene glycol and maize grain supplementation on dry matter intake (DMI). ^a,b^ Statistically significant differences between groups are indicated by different letters (*p* ≤ 0.05) ± standard deviation. TG—2.5 kg triticale grain/cow per day supplemented from 14 days prepartum to day 56 postpartum, PG—2.5 kg triticale grain/cow per day supplemented from day 14 before parturition to day 56 postpartum, and 400 g propylene glycol/cow per day from 14 days prepartum to 14 days of lactation, MG—2.5 kg maize grain/cow per day supplemented from day 14 before parturition to day 56 postpartum.

There were no significant differences between the groups in mean dry matter intake from −21 day to 56 day of lactation (*p* > 0.05) (Figure 1).

Propylene glycol and maize grain had an effect on the hematic glucose concentration at 28 day of lactation that resulted significantly higher compared to TG group. (*p* ≤ 0.05; Table 3).

**Figure 2:**
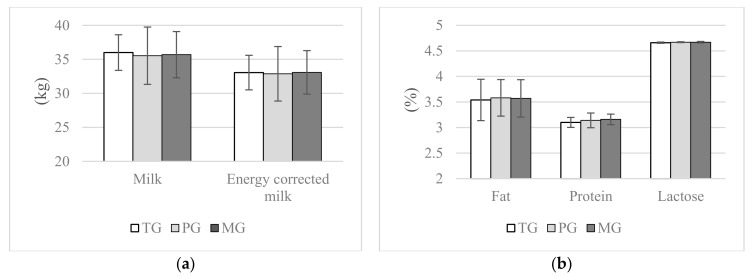
Effect of propylene glycol and maize grain supplementation on mean milk performance, (**a**) milk and energy corrected milk yield and (**b**) milk composition. ^a,b^ Statistically significant differences between groups are indicated by different letters (*p* ≤ 0.05) ± standard deviation. TG—2.5 kg triticale grain/cow per day supplemented from 14 days prepartum to day 56 postpartum, PG—2.5 kg triticale grain/cow per day supplemented from day 14 before parturition to day 56 postpartum, and 400 g propylene glycol/cow per day from 14 days prepartum to 14 days of lactation, MG—2.5 kg maize grain/cow per day supplemented from day 14 before parturition to day 56 postpartum.

Cows assigned to the PG and MG groups had significantly higher cholesterol concentrations during lactation, this was observed between both experimental groups and TG in 14 DIM, instead at 28 and 56 DIM difference was significant only between the MG and the TG group (*p* ≤ 0.05). Propylene glycol and maize grain had no effect on triglyceride concentrations during the transition period (*p* > 0.05); nevertheless, at 56 d lower blood triglyceride concentrations were recorded in cows assigned to PG (*p* ≤ 0.1) and MG (*p* ≤ 0.05) compared with the TG group. Both treatments, propylene glycol and maize grain, had no effect on NEFA and BUN concentrations as well as ASPAT activity in blood (*p* > 0.05). No significant differences were found between the groups in terms of mean milk yield and mean energy corrected milk production (*p* > 0.05, Figure 2). Neither propylene glycol nor maize grain had an effect on fat, protein and lactose contents in milk up to 56 DIM (*p* > 0.05, Figure 2).

Propylene glycol and maize grain had a statistically confirmed effect on improving the fertility parameters of cows. Shortening of the period to first ovulation was found in cows assigned to the PG group (*p* ≤ 0.05, Table 4). Higher first service conception rates and fewer services per conception were recorded in cows from the PG and MG groups in comparison to the TG group (*p* ≤ 0.05, Table 4).

## 4. Discussion

Decreased fatty acid mobilization from adipose tissue as well as their complete oxidation in the liver seem to be the main objective during early lactation of dairy cows. Some energy supplement and glucogenic additives were described as good approaches to improve the negative energy balance [14,35,36]; however, literature sources lack data on their comparisons. Probably propylene glycol and/or maize grain contain by–pass starch, which when digested to glucose in the small intestine has a better glucogenic effect compared to excessive rumen degradation of starch, which is the hypothesis of the present study.

Despite the fact that at the beginning of the close-up period the animals were in better body condition, the mobilization of body reserves was moderate and no differences of BCS and it changes were detected between the groups. These results are in correspondence with moderate NEFA concentrations without statistical differences between the groups. Similar results on the lack of the PG effect on blood NEFA concentration were shown by Piantoni and Allen [7]. A different observation on the positive effect of PG on a decrease in lipolysis and reduction of blood NEFA concentration was presented by Rizos et al. [37]. Moreover, a positive effect of starch digestion in the small intestine on a decrease in NEFA levels was reported by Knowlton et al. [38] and Lemosquet et al. [39], who confirmed that “by-pass” starch results in a reduction of lipolysis. In turn, Lykos et al. [40] claimed that blood NEFA concentration is decreased at an increase of starch fermentation in the rumen. However, Garnsworthy et al. [26] noted that the site of starch breakdown, rumen microbial degradation or small intestine digestion had no effect on blood NEFA levels. Both treatments had no effect on ASPAT activity concentration; however, this marker activity in blood of all cows was below 100 U/L and indicated hepatic health [41] while also confirming a moderate energy deficit. Both propylene glycol and maize grain had a positive effect on increasing the blood glucose concentration at 28 d after calving. Despite a suggestion that blood glucose concentration as a metabolic status index is questionable [42], it could confirm a more effective glucogenic influence of PG and MG in comparison to excessive rumen degradation of starch from TG. This finding is consistent with the results of Nielsen and Ingvartsen [43], who stated that propylene glycol could increase blood glucose level, similarly as it was proposed by Reynolds [44]. A positive effect of propylene glycol on an increase in blood glucose concentration was noted by Liu et al. [13], Adamski et al. [14], Piantoni and Allen [7]. In turn, Chibisa et al. [45], Chung et al. [46] and Lomander et al. [47] observed no statistically confirmed influence of PG on this blood marker. In turn, a positive effect of by–pass starch digested in the small intestine on increased blood glucose levels was confirmed by Lemosquet et al. [39], whereas Garnsworthy et al. [26] observed no effect of starch digestion site on blood glucose concentration.

Many authors claimed [37,48] that severe negative energy balance in the early lactation had negative effect on follicular development, oocyte competence, embryo survival and in consequence decreased reproduction performance. Reist et al. [33] noted a relationship between negative energy balance on the interval from calving to first ovulation. In turn, Chapinal et al. [49] claimed that a negative energy balance and elevated NEFA and BHBA levels are connected with impaired reproductive performance; however, the mechanism underlying the effect of a negative energy balance during the transition period on fertility some weeks later is unclear. Leroy et al. [50] showed a direct toxic effect of NEFA and BHBA on in vitro oocyte maturation.

Despite moderate energy deficit both treatments (PG and MG) had a positive effect on fertility parameters such as shortening the period to first ovulation, higher first services conception rate and lower services per conception. Shrestha et al. [51] argued that a delayed first ovulation is one of the most common ovarian dysfunction in high yielding dairy cows. Thus a reduced period to first ovulation by 13.6 and 7.9 days in the PG and MG groups, respectively, compared to the TG group could confirm the influence of propionate from propylene glycol, as well as small intestine digestive starch from maize grain as a glucose precursor. Similar results shortening the interval from calving to first estrus after administration of 600 mL of propylene glycol as the oral dose during the first week after calving were observed by Borş et al. [36]. Additionally, a better first service conception rate and first services per conception, which were observed in the PG and MG groups could confirm that both glucogenic additives influence oocyte and embryo quality [2]. It is known that blood cholesterol level is positively related to energy intake [52], thus the higher cholesterol concentration observed in the PG and MG groups during lactation in the present study confirmed this thesis. Mohebbi-Fani et al. [53] showed the importance of cholesterol in the reproductive hormone synthesis; in turn, Rabiee and Lean [52] suggested that cholesterol uptake into ovarian cells may be promoted by glucose. Additionally, Reist et al. [33] claimed that cholesterol concentration could have a strong influence on the interval from calving to conception. Therefore higher blood glucose and cholesterol concentrations in the PG and MG groups were connected with the statistically confirmed better fertility parameters.

Lowered energy uptake in early lactation have an effect on an inferior metabolic status of cows, which is connected with a longer interval to first ovulation [48]. In the present study voluntary dry mater intake was similar during the close–up period and the first two months of lactation. This result may confirm better utilization of propionate from propylene glycol, as well as small intestine digestive starch from maize grain as a glucose precursor.

The treatments (PG, MG) were found to have no effect on milk yield, energy corrected milk production or milk composition. Probably similar milk performance results may show that homeostasis of dairy cows from all the groups was maintained. Replacement of 2.5 kg of triticale grain containing starch excessively degraded in the rumen with 2.5 kg maize grain with by–pass starch, which is digested in the small intestine, as well as supplementation of 400 g of propylene glycol were insufficient to influence milk performance. Similar results showing no effect on the milk yield were reported after 600 g of propylene glycol administration as an oral dose during the first week after calving [36] and 200 g of propylene glycol administration [54]. Additionally, Chung and al. [54] found no PG effect on milk content without lactose.

## 5. Conclusions

In conclusion, both treatments of dairy cows with PG and MG improved fertility parameters, while they had a slight effect on the metabolic profile and no effect on milking performance in dairy cows. Fertility parameters are strongly connected with reproductive performance, which is a key factor affecting profitability of milk production. Thus PG and MG could be reconsidered in the close–up and fresh diets.

## Figures and Tables

**Table 1:** Ingredients and nutrient composition of the experimental total mixed ration (TMR) diets.

Items	Close-Up		Lactation	
	Triticale	Maize	Triticale	Maize
Ingredients, % DM				
maize silage	38.2	38.2	31.2	31.2
alfalfa silage	16.7	16.7	13.7	13.7
grass silage	7.8	7.8	5.6	5.6
sugar beet pulp silage	7.6	7.6	5.8	5.8
soybean meal	4.4	4.4	5.3	5.3
rapeseed meal	2.3	2.3	2.1	2.1
triticale grain	11.4	---	8.7	---
maize grain	---	11.4	---	8.7
barley grain	4.9	4.9	22.7	22.7
wheat straw	4.7	4.7	3.6	3.6
minerals and vitamins	2.0	2.0	1.3	1.3
Nutrient concentration, in 1 kg of DM				
UFL	0.84	0.85	0.87	0.88
PDIN (g)	83	83	88	88
PDIE (g)	82	82	89	89
Ca	7.28	7.23	8.23	8.19
P	3.59	3.46	3.02	2.95
LFU	0.87	0.87	0.72	0.72

DM—dry matter; UFL—feed unit for lactation (1700 kcal NE_L_); PDIN—dietary protein undegraded in the rumen, but truly digestible in the small intestine and microbial protein, which could be synthesized in the rumen from degraded dietary nitrogen, when energy and other nutrients are not limiting; PDIE—dietary protein undegraded in the rumen, but truly digestible in the small intestine and microbial protein, which could be synthesized in the rumen from the energy available in the rumen, when degraded nitrogen and other nutrients are not limiting, LFU—feed unit for lactating dairy cows, VDMI—voluntary dry matter intake.

**Table 2:** Effect of propylene glycol and maize grain supplementation on body condition score (BCS).

Group	Days from Calving					BCS Changes			
	−21 Day	0	+14 Day	+56 Day	−21→0	0→14	14→56	0→56	−21→56
TG	3.69	3.54	3.42	3.31	−0.15	−0.13	−0.11	−0.23	−0.39
PG	3.75	3.58	3.42	3.29	−0.17	−0.17	−0.13	−0.29	−0.46
MG	3.73	3.54	3.48	3.35	−0.19	−0.06	−0.13	−0.19	−0.38
SEM	0.022	0.017	0.017	0.023	0.024	0.022	0.027	0.030	0.032

TG—2.5 kg triticale grain/cow per day supplemented from 14 days prepartum to day 56 postpartum, PG—2.5 kg triticale grain/cow per day supplemented from day 14 before parturition to day 56 postpartum, and 400 g propylene glycol/cow per day from 14 days prepartum to 14 days of lactation, MG—2.5 kg maize grain/cow per day supplemented from day 14 before parturition to day 56 postpartum.

**Table 3:** Effect of propylene glycol and maize grain supplementation on blood serum metabolites.

Blood indices	Group	Time of Sample Collection				
		−21 Days	−7 Days	+14 Days	+28 Days	+56 Days
Glucose (mmol/L)	TG	3.65	3.56	2.41	2.40 ^a^	2.64
	PG	3.68	3.63	2.54	2.60 ^b^	2.74
	MG	3.64	3.54	2.50	2.70 ^b^	2.58
	SEM	0.021	0.025	0.027	0.026	0.022
NEFA (mmol/L)	TG	0.126	0.177	0.279	0.220	0.188
	PG	0.151	0.185	0.269	0.190	0.179
	MG	0.145	0.195	0.257	0.207	0.160
	SEM	0.009	0.009	0.012	0.009	0.007
Cholesterol (mmol/L)	TG	3.48	1.95	1.72 ^a^	3.53 ^a^	4.32 ^a^
	PG	2.89	1.86	2.83 ^b^	3.91 ^a,b^	5.02 ^ab^
	MG	2.60	1.73	2.54 ^b^	4.55 ^b^	5.46 ^b^
	SEM	0.103	0.069	0.115	0.143	0.130
Triglycerides (mmol/L)	TG	0.292	0.272	0.187	0.171	0.135 ^a^
	PG	0.296	0.257	0.177	0.134	0.164 ^b^
	MG	0.283	0.243	0.170	0.160	0.205 ^b^
	SEM	0.008	0.009	0.005	0.008	0.008
ASPAT (U/L)	TG	53.5	55.0	82.1	61.9	56.3
	PG	44.0	47.2	82.5	69.9	58.9
	MG	54.0	51.2	79.1	61.3	61.8
	SEM	1.58	2.02	3.52	2.04	1.73
BUN (mmol/L)	TG	2.31	3.45	2.56	2.03	1.50
	PG	2.76	3.25	2.52	1.52	1.18
	MG	2.24	3.41	2.60	1.65	1.22
	SEM	0.119	0.224	0.172	0.079	0.072

^a,b^ Statistically significant differences between groups are indicated by different letters (*p* ≤ 0.05). TG—2.5 kg triticale grain/cow per day supplemented from 14 days prepartum to day 56 postpartum, PG—2.5 kg triticale grain/cow per day supplemented from day 14 before parturition to day 56 postpartum, and 400 g propylene glycol/cow per day from 14 days prepartum to 14 days of lactation, MG—2.5 kg maize grain/cow per day supplemented from day 14 before parturition to day 56 postpartum.

**Table 4:** Effect of propylene glycol and maize grain supplementation on fertility parameters.

Group	Days to First Ovulation	First Service Conception Rate	Services Per Conception	Days Open
TG	41.5 ^a^	0.57 ^a^	1.61 ^a^	117
PG	27.9 ^b^	0.67 ^b^	1.34 ^b^	105
MG	33.6 ^a,b^	0.73 ^b^	1.26 ^b^	108
SEM	1.236	0.055	0.094	3.533

^a,b^ Statistically significant differences between groups are indicated by different letters (*p* ≤ 0.05). TG—2.5 kg triticale grain/cow per day supplemented from 14 days prepartum to day 56 postpartum, PG—2.5 kg triticale grain/cow per day supplemented from day 14 before parturition to day 56 postpartum, and 400 g propylene glycol/cow per day from 14 days prepartum to 14 days of lactation, MG—2.5 kg maize grain/cow per day supplemented from day 14 before parturition to day 56 postpartum.
